# Exploring the interplay between circadian rhythms and obesity: A Boolean network approach to understanding metabolic dysregulation

**DOI:** 10.1371/journal.pone.0331218

**Published:** 2025-09-09

**Authors:** Meitner Cadena, George E. Barreto

**Affiliations:** Department of Biological Sciences, University of Limerick, Limerick, Ireland; Tabriz University of Medical Sciences, IRAN, ISLAMIC REPUBLIC OF

## Abstract

This study investigates the interaction between circadian rhythms and lipid metabolism disruptions in the context of obesity. Obesity is known to interfere with daily rhythmicity, a crucial process for maintaining brain homeostasis. To better understand this relationship, we analyzed transcriptional data from mice fed with normal or high-fat diet, focusing on the mechanisms linking genes involved with those regulating circadian rhythms. We performed biological enrichment analysis and Boolean network modeling to identify direct interactions between these genes. The resulting mathematical model provided a comprehensive system of gene interactions, primarily highlighting lipid metabolism. Our findings revealed key insights into the effects of obesity on circadian rhythm genes, particularly the under-expression of core genes such as *Bmal1* and *Clock*. Crucially, we identified a reciprocal interaction between obesity and circadian genes, where disruptions on one exacerbated the dysfunction in the other. This mechanism suggests that the disruption of circadian rhythms plays a pivotal role in worsening the metabolic disturbances associated with obesity, providing new perspectives for targeting circadian pathways in obesity-related metabolic disorders.

## 1. Introduction

Obesity is a modern disease primarily associated with lifestyle factors. Sedentary work activities, combined with diets rich in ultra-processed foods that are often consumed in larger quantities due to their competitive cost and palatability, have contributed to metabolic balances, leading to excess weight accumulation. This surplus body mass, which is disproportionately high relative to an individual’s weight, is reflected in components of the human body that have increased in size to maintain functionality [1].

Obesity typically leads to low-grade chronic inflammation in the body, which can contribute to the development of various comorbidities. This type of inflammation is characterized by the secretion of proinflammatory cytokines, such as interleukin-6 (IL-6) and tumor necrosis factor-alpha (TNF-α), which have systemic effects throughout the body [2]. One of the major consequences of this inflammation is the emergence of metabolic disorders, particularly metabolic syndrome [3,4]. This condition is diagnosed when at least three of the following markers are present: abdominal obesity, high blood pressure, elevated fasting blood glucose, high triglycerides, and low HDL cholesterol [5]. Metabolic syndrome is a key indicator of the increased risk for serious chronic diseases, including those related to blood sugar dysregulation and cardiovascular health [6]. These include type 2 diabetes and insulin resistance, as well as atherosclerosis. At the brain level, this persistent inflammation activates the brain’s immune system, particularly glial cells, which produce proinflammatory cytokines such as TNF- α, IL-1β, and IL-6 [7–9]. This chronic inflammation can accelerate brain aging by up to 10 years compared to healthy individuals [10], which can lead to critical structural changes such as reduced cortical thickness [11–15]. These changes in the cortex can result in cognitive and functional impairments, including deficits in sensory perception, motor control, memory, language, communication, and emotional regulation [16].

Circadian rhythms, which regulate metabolic, physiological and behavioral coordination activities in 24-hour cycles, have a bidirectional relationship with obesity [17,18]. Obesity can disrupt these rhythms, for instance, by altering hormone production necessary for food metabolism, which may lead to insulin resistance, or by fragmenting and reducing sleep quality, impairing the coordination of energy expenditure with appetite [19]. Additionally, eating at irregular times promotes a metabolic state oriented toward caloric storage, contributing to weight gain and, ultimately, obesity [20]. This complex interplay between circadian rhythms further worsens the metabolic imbalance, as disrupted circadian rhythms become an intrinsic factor that perpetuates obesity [21]. Moreover, obesity can be influenced by external factors that disrupt circadian rhythms, such as eating late at night, exposure to artificial light, night-shift work, or even epigenetic modifications that transmit these altered rhythms [22,23].

Despite substantial progress in characterizing obesity’s pathophysiology, how it perturbs the body’s internal clock remains poorly understood [24]. To address this gap, we employ Boolean network modeling to capture- and predict- the dynamic interactions between circadian regulators and metabolic genes under both normal and high-fat diet conditions. In a Boolean framework, each gene or protein is represented as either “on” or “off”, and its state at each time step is determined by simple logical rules that helps identify conditions under which different evolutionary paths may emerge [25]. This abstraction helps us to simulate complex system behavior and identify critical points at which a high-fat diet can drive the network toward dysregulated metabolic states. Furthermore, we focus our analysis on the cerebral cortex, given evidence that obesity disrupts cortical metabolism and redox balance, with downstream effects on neurobehavioral function and glial-neuronal signalling [26–35]. Although Boolean networks have proven useful in isolated studies of metabolic regulation, no prior work has integrated these approaches to map how obesity reshapes circadian-metabolic crosstalk. By constructing and simulating a Boolean model of cortex-derived transcriptomic data, we aim to reveal the system-level mechanisms by which obesity disturbs both clock genes and lipid metabolism pathways, thereby uncovering potential intervention points for restoring metabolic homeostasis.

## 2. Materials and methods

### 2.1. Murine brain datasets

We utilized a publicly available transcriptome dataset from the National Center for Biotechnology Information (2024) with accession number GSE179711. This dataset was previously analyzed to investigate the roles of non-coding RNAs in regulating cell proliferation and differentiation, neurotransmission, and neuronal excitability in the context of obesity [36]. The dataset captures genetic alterations in four male mice exposed to a high-fat diet (HFD) for 8 weeks, compared to four control mice on a normal diet (ND). Transcriptomes were collected from the brain cortex of 16-week-old mice, allowing for a direct comparison of diet-induced genetic changes.

### 2.2. Data preparation

Transcriptomes were assembled using the align function from the DESeq2 package [37] in the R programming language [38]. To ensure comparability, transcriptomes were normalized to account for variations due to sequencing depth, batch effects, and differences in cellular compositions [39]. This essential step allowed us for reliable comparisons across samples under similar conditions, as well as between groups exposed to different conditions. Next, differentially expressed genes (DEGs) were then identified using the limma package [40], with a significant threshold set at a p-value of 0.05. These DEGs were visualized using heatmaps, generated by the hmReady function from the ggdendroplot package [41], and volcano plots created with the EnhancedVolcano function from the EnhancedVolcano package [42].

#### 2.2.1. Enrichment analysis.

A DAVID-based enrichment analysis [43] was conducted separately for up- and down-regulated genes, focusing on gene ontology (GO) terms: biological processes (BP), cellular components (CC), and molecular functions (MF). From the top functional annotations, we identified DEGs that were common to BP, CC, and MF categories, indicating potential functional similarities [44]. To further explore gene interactions, we analyzed these up- and down-regulated DEGs through interaction networks (PPIs) to identify clusters with more cohesive structures using the Metascape tool [45]. Metascape applies the molecular complex detection (MCODE) algorithm to pinpoint densely connected within PPIs, with region representing a functional cluster. PPIs were sourced from multiple databases: STRING, which compiles known and predicted interactions and scores them to form reliable networks [46]; BioGrid, which emphasizes biological interactions like PPIs and genetic links [47]; and OmniPath, which includes diverse interactions such as PPIs and miRNA-mRNA [48]. These networks enabled comprehensive comparative and pathways enrichment analyses.

#### 2.2.2. Boolean networks.

The initial analysis generated undirected gene networks, meaning relationships between gene pairs were ambiguous, and causality – if present- was undefined. To address this limitation, we applied the GENIE3 algorithm [49] specifically to a network of genes associated with circadian rhythms. GENIE3 enables the construction of Boolean networks, where nodes are governed by Boolean functions that establish directed, causal relationships among genes. Since this type of network represents genes as either “on” (active) or “off” (inactive) at any given time, the Boolean functions define simple logical rules that indicate states of the genes with which they interact. This structure allowed us to simulate the network’s evolution, predicting the activation and inactivation of nodes based on initial conditions [50]. While Boolean networks provide a simplified view by treating nodes as either fully activated or inactivated, real biological interactions often involve partial activation states. More complex models, such as probabilistic networks or those based on differential equations [51,52], may capture this nuance more effectively. However, Boolean networks offer an interpretable approximation of potential system states, making them valuable for preliminary insights. The GENIE3 algorithm employs random forests [53] to simulate gene knockouts, estimating the strengths of previously undirected relationships. By setting appropriate thresholds on these estimates, the algorithm determines directed connections between genes. Since its development, GENIE3 has been widely applied to gene network inference [54–56]. Finally, to understand the genetic impacts of recurrent high-fat food consumption`, we simulated the resulting Boolean network using the getAttractors function from the BoolNet package [57]. We implemented a synchronous network model, allowing multiple nodes to change states simultaneously at each step, accounting for all possible combinations of Boolean values that nodes may assume [58]. This approach facilitated the identification of stable network states, providing insights into how circadian rhythm genes respond to high-fat diets.

## 3. Results

The transcriptome data were processed to construct a Boolean network. S1 Fig illustrates key elements of the normalization process and some of its outcomes. In S1A Fig, the normalization effect is displayed, demonstrating comparable read counts across samples, particularly in median Q2 and upper quartile (Q3) statistics. S1B Fig presents the relationship between the observed means and standard deviations of analyzed genes. By fitting nonparametric models to describe the median trend within this scatter plot, we can assess the statistical significance of the analyzed genes.

**Fig 1:**
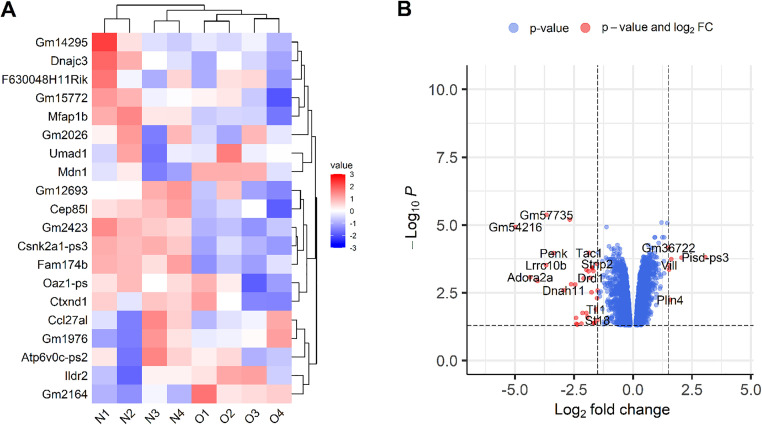
Features of detected DEGs. (A) Variation in gene expression levels between the samples subjected to normal diet (ND) or high-fat diet (HFD). (B) Distribution of genes based on log fold change and log p-value.

Following the identification of differentially expressed genes (DEGs), Fig 1A highlights the 20 most statistically significant genes, categorized by their up- and down-regulated states in response to the dietary shift between normal diet (ND) and high-fat diet (HFD). This heatmap shows gene expression patterns for ND samples (left) and HFD samples (right). Although expression levels vary within these groups, overall trends emerge. For example, *Gm14295* exhibits reduced expression in response to HFD, while *GM2164* shows increased expression. Fig 1B displays a scatter plot of the DEGs, with the log transformations of fold change and p-value. Only genes with p-values less than 0.05 were included. Vertical reference lines are shown at log fold change of −1.5 and 1.5. In this plot, genes marked in red are considered significant, meeting the criteria of a p-value below 0.05 and a log fold change outside ±1.5. Genes with a negative log fold change are classified as down-regulated, while those with a positive log fold change are classified as up-regulated.

### 3.1. Gene enrichment analysis

**Fig 2:**
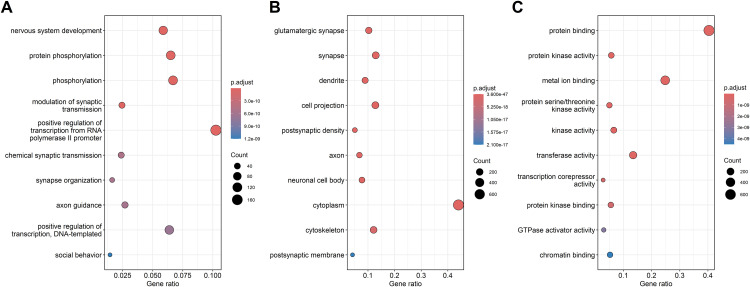
Top 10 significant functional annotations of up-regulated DEGs. (A) Biological processes, (B) cellular components, and (C) molecular functions, ranked by adjusted p-values. Functional annotations are ordered by decreasing significance, with color indicating significance according to the legend’s color scale, the ratio of genes on the horizontal axis, and DEG count represented by circle size.

**Fig 3:**
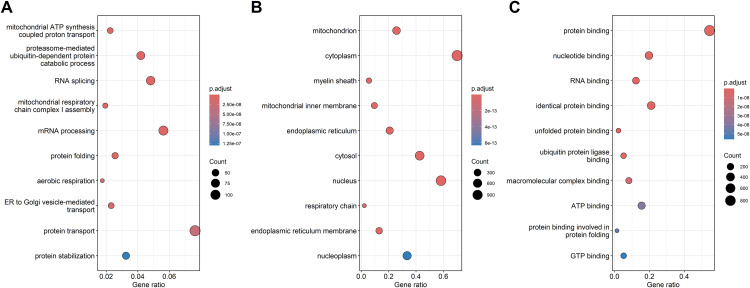
Top 10 significant functional annotations of down-regulated DEGs. (A) Biological processes, (B) cellular components, and (C) molecular functions, ranked by adjusted p-values. Functional annotations are ordered by decreasing significance, with color indicating significance level based on the legend’s color scale, the ratio of genes on the horizontal axis, and DEG count represented by circle size.

The enrichment analysis identified the ten most significant functional annotations within the gene ontology categories BP, CC and MF. These are presented in Fig 2 for up-regulated DEGs and Fig 3 for down-regulated DEGs. Notably, for up-regulated DEGFs, the most significant functional annotations are nervous system development in BP and glutamatergic synapse in CC. These are distinguished by their markedly low p-values compared to other GO annotations within the same type of category, indicating strong enrichment. Interestingly, such distinctive functional annotations are absent when analyzing MF for up-regulated DEGs, as well as PB, CC and MF for down-regulated DEGs, suggesting unique functional shifts associated primarily with up-regulated DEGs in response to dietary change.

**Fig 4:**
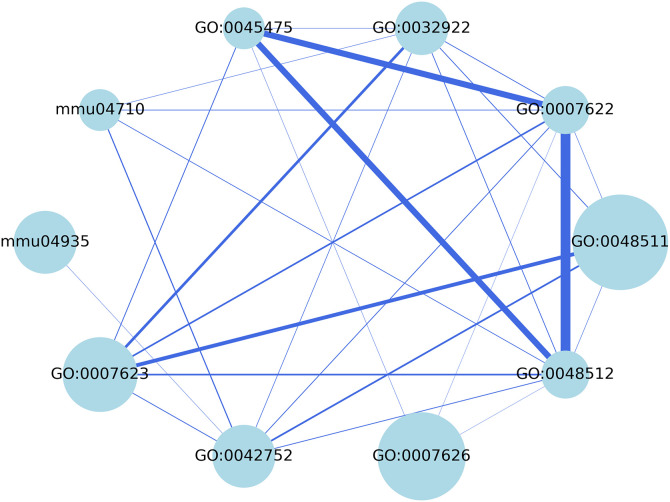
Network of functional annotations related to circadian rhythms for upregulated DEGs. Nodes represent functional annotations, with node size indicating the number of genes within each Edges represent relationships between nodes, with edge thickness reflecting the strength of the association.

Further genes were incorporated into the analysis using the Metascape tool, which generated a network of functional annotations related to circadian rhythms, shown in Fig 4 with up-regulated DEGs included. Detailed descriptions and types of nodes within this network are detailed in Table 1. No relevant clusters were identified among the down-regulated DEGs in terms of circadian rhythms, except for the functional annotation WP544, which is associated with circadian rhythms by physical exercise. This network includes annotations not only from BP, CC and MF, but also pathways such as mmu04710 and mmu04935. Each node represents a functional annotation, with node size indicating the number of genes within that annotation. Notably, GO:0048511 contains the highest number of genes among all annotations, followed closely by GO:0007626. The former is related to rhythmic behavior, and the latter to rhythmic process. Edges, which represent interactions between genes in different functional annotations, are weighted according to the scores by Metascape, and are visually reflected by edge thickness – the thicker the edge, the stronger the relationship. For instance, the connection between GO:0007622 and GO:0048512 is the strongest on this network. It is important to note that mmu04935 and GO:0007626 are not directly related to circadian rhythms but were included because they bridge the network shown in Fig 4 with other networks.

**Table 1:** Description of functional annotations related to circadian rhythms when considering up-regulated DEGs.

Functional annotation	Type	Description
GO:0048511	GO	Rhythmic process
GO:0007626	GO	Rhythmic behavior
GO:0032922	GO	Circadian regulation of gene expression
GO:0045475	GO	Locomotor rhythm
mmu04710	KEGG pathway	Circadian rhythm – Mus musculus (house mouse)
mmu04935	KEGG pathway	Growth hormone synthesis, secretion and action – Mus musculus (house mouse)
GO:0007623	GO	Circadian rhythm
GO:0042752	GO	Regulation of circadian rhythm
GO:0007626	GO	Locomotory behavior
GO:0048512	GO	Circadian behavior

### 3.2. Building a Boolean network

**Fig 5:**
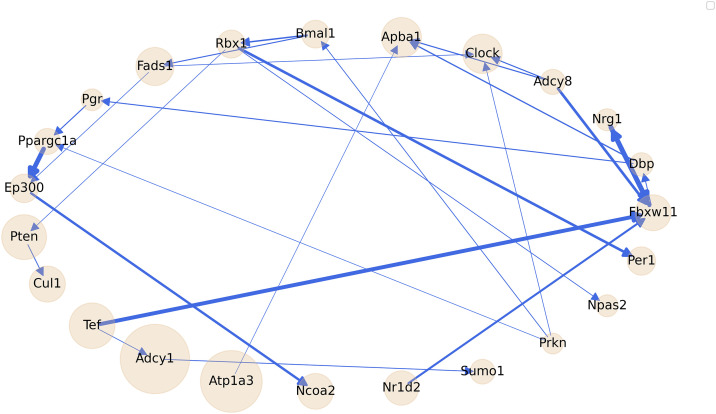
Boolean network of circadian rhythms and their interplay with metabolism-related genes under normal diet conditions. Nodes represent individual genes, with node size corresponding to the log-average of normalized transcriptome counts for each gene. Arrows indicate regulatory interactions, with their thickness representing the strength of influence from one gene to another.

The network shown in Fig 4 combined with genes present in WP544 and mmu04710 resulted in a set of 87 genes. Using the GENIE3 algorithm, we derived a direct network under ND conditions (Fig 5), which comprises 23 genes. In this network, node sizes represent the log average of normalized transcriptome counts under ND conditions, while edge weights – calculated through the GENIE3 algorithm – and graphically represented by the thickness of the edge indicate the strength of regulatory interactions. This Boolean network model represents complex biological interactions as events ‘on/off’ for gene activity, allowing us to simulate how HFD disrupts metabolic pathways. This network integrates various pathways that interact to regulate key biological processes. One prominent is circadian regulation, primarily driven by genes such as *Bmal1*, *Clock* and *Npas2*, which orchestrate metabolic processes in 24-hour cycles to maintain energy homeostasis [59,60]. Within this network, energy metabolism is a key pathway led by *Ppargc1a*, a gene crucial for mitochondrial biogenesis, oxidative metabolism, energy expenditure and adaptation to conditions like physical exercise, fasting or high-fat diets [61]. Another major pathway is lipid metabolism, represented by *Fads1*, which plays a role in the biosynthesis of polyunsaturated fatty acids, thereby affecting cell membrane fluidity and influencing signaling in metabolism and inflammation pathways [62,63].

In addition to these metabolic pathways, the network includes genes involved in signal transduction such as *Adcy1* and *Adcy8*, which support cellular communication and responses to external factors like hormones, nutrients, and environmental changes [64]. It also features genes central to transcriptional regulation, including *Ep300* and *Ncoa2*, which adjust gene expression in response to internal and environmental changes [65,66]. Finally, genes such as *Pten* are involved in cell growth and survival, as they regulate the PI3K/Akt signaling pathway to maintain cellular homeostasis [67].

Table 2 presents key properties of the resulting network, calculated using functions from the igraph package [68]. The “hub” metric reflects a node’s influence within the network, measured by its degree – i.e., the number of connections a node has. Accordingly, the genes *Npas2*, *Per1* and *Pten* appear to exert strong influence over other genes in the network. Edge density indicates the proportion of existing edges compared to the maximum possible number of edges in the network. Its observed value is low (0.026), indicating that the network is sparse, as expected in biological systems where not all genes interact directly. Closeness measures how close a node is to all other nodes, capturing how information can travel from this node across the network. Its moderate value (0.301) suggests that the hubs can effectively influence the broader network. Betweenness quantifies the number of shortest paths that go through a node, highlighting nodes that serve as key connectors. Its high value (0.826) indicates that the hubs may function as critical connectors or bottlenecks for information flow within the network. Lastly, entropy evaluates the distribution of degrees in the network, where a value greater than 1 suggests a high level of disorder, indicating that some genes are significantly more influential than others. This parameter reflects that the analyzed biological regulatory network is organized yet complex.

**Table 2:** Network properties of the Boolean network achieved.

Genes identified as hubs	Degree*	Edge density	Closeness*	Betweenness*	Entropy
*Npas2*, *Per1*, *Pten*	1.130	0.026	0.301	0.826	1.280

(*) Mean of the parameter.

### 3.3. Simulation of the Boolean network

The Boolean network constructed in the previous subsection was simulated to explore its potential evolutionary states. This evolution involves generating random Boolean networks, where the nodes switch between active and inactive states. Each time a node switches its Boolean value, the network state changes, and the system evolves towards attractors – stable states that are not expected to change over time. The simulated Boolean network will eventually converge to one or more attractors, each with a certain probability of being reached. This process allowed us to track the convergence of a set of interacting genes under complex regulatory schemes [69].

**Fig 6:**
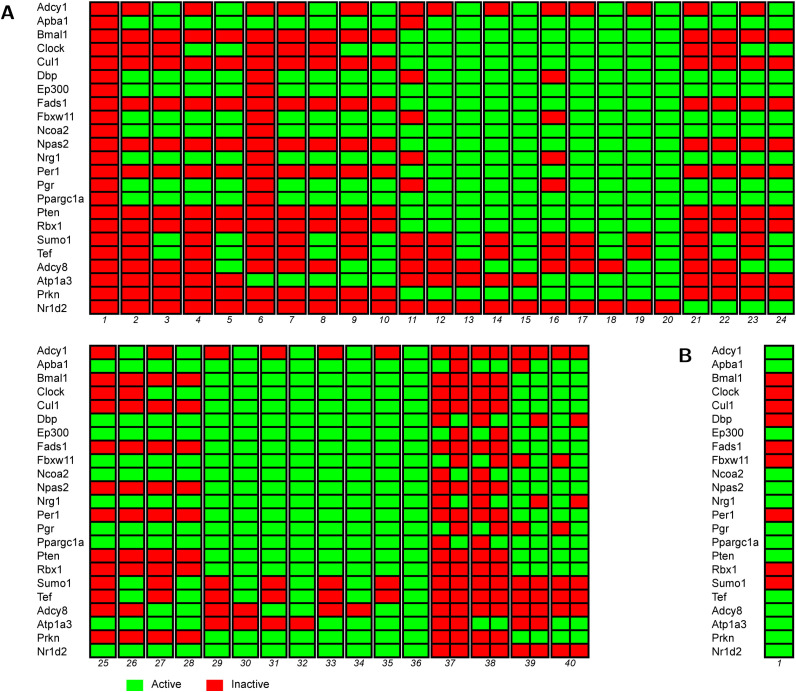
Simulation of the Boolean network on circadian rhythms under (A) normal diet and (B) high fat diet conditions. Genes are shown on the vertical axis, with their states of evolution on the horizontal axis. The final states represent the attractors reached.

Fig 6A displays the attractors achieved by the developed Boolean network under ND conditions. This figure reveals that the network can converge to 40 distinct attractors, depending on the initial Boolean values of the genes. Notably, the last four attractors demonstrate two possible evolutionary paths, leading to Hopf bifurcations. These bifurcations result in oscillatory behaviors, where the network alternates between at least two states [70]. In our cases, these cycles involve two distinct states.

As an example, the attractor 40 whose limit state oscillates between two different states, forms an attractor with two states. In these states, all genes maintain the same Boolean values except for *Dbp*, Fbxw11, *Nrg1*, and *Prg*. S2 Fig illustrates the behavior of these exceptions. The figure shows iterations of all these genes together, regardless of their initial Boolean values. For instance, these genes are initially set to Active, Inactive, Active, and Active, respectively. After one iteration, the network reaches the expected states. It is noteworthy that each expected state follows directly from the previous one, without the need for further iterations. This oscillation between states generates an attractor that is governed by a Hopf bifurcation.

Next, we perturbed the developed Boolean network to reflect HFD-related conditions. For this, we consider the up-regulated and down-regulated DEGs. As shown in Fig 6A, all genes except *Per1* are either up- or down-regulated. For the up-regulated genes, we set their initial condition to 1 to simulate over-expression, while for down-regulated genes, we set their initial condition to 0 to simulate knockout. However, some genes do not need to be pre-set due to the network’s intrinsic dynamics. In fact, the network’s structure and behavior allow certain expected results to emerge naturally, without requiring prior adjustment. After conducting preliminary simulations, it was determined that only the following genes needed to be fixed: *Tef*, *Adcy8*, *Atp1a3*, *Prkn*, *Nr1d2*, *Fbxw11*, *Nrg1*, *Pgr*, *Sumo1*, *Bmal1*, *Pten*, *Npas2*, *Cul1* and *Clock*. Fig 6B shows the evolution and convergence of the perturbed network. This time, the network converged to a single attractor, achieved through a unique state. In this attractor, *Per1* is downregulated. This result suggests a possible scenario for how part of the circadian rhythm genetic system may behave under a HFD, once the system stabilizes into an obesity-related gene activity pattern. Such a dynamic model could provide valuable insights for future research and potential therapeutic strategies.

## 4. Discussion

In this study, we constructed a biological representation of circadian rhythms using Boolean networks, offering a novel integrative approach to understanding their complex biological functions. Through this mathematical modelling framework, we were able to elucidate key interactions between circadian rhythms and various metabolic processes, specifically in the context of obesity. Importantly, the model highlights the bidirectional interaction between circadian rhythms and obesity, revealing how this metabolic dysfunction can perpetuate a cycle of regulatory imbalances.

Lipid metabolism is a crucial area of interest when addressing the implications of obesity. Notably, the Boolean network developed in this study highlights genes directly involved in lipid metabolism, revealing a strong connection between circadian rhythms and lipid homeostasis. For instance, *Ppargc1a* plays a key role in pathways related to lipid metabolism, including fatty acid oxidation and mitochondrial biogenesis [71]. *Fads1* is involved in the biosynthesis of unsaturated fatty acids and the regulation of cell membrane fluidity [72], while *Adcy1* and *Adcy8* participate in cAMP signaling pathways that influence lipid metabolism by modulating fatty acid synthesis in response to AMP/ATP ratios [73]. Additionally, *Ncoa2* mediates the co-activation of genes involved in lipid metabolism (Rollins et al., 2015). These metabolic processes occur in the context of key circadian rhythms genes such as *Clock*, *Bmal1*, *Dbp*, *Per1*, *Npas2*, and *Nr1d2*, highlighting the critical role of circadian rhythms in regulating lipid metabolism. Specifically, *Clock* and *Bmal1* form a heterodimer that enhances the expression of *Ppargc1a*, promoting fatty acid oxidation and mitochondrial biogenesis in response to increased energy demands during activities like fasting or physical exercise, which mainly correspond to active phase of the body during the day [74].

Our findings show that other circadian genes, such as *Dbp*, *Per1*, *Npas2* and *Nr1d2*, have more specific roles. *Dbp* increases its levels at the start of the active phase to activate Ppargc1a, responding to energy demand spikes [75]. *Per1* down-regulates the *Clock*-*Bmal1* complex at specific times during the active phase, when its activity is less needed [76]. *Npas2* interacts with *Ppargc1a* in a positive feedback loop, enhancing lipid metabolism to meet energy demands [77]. In contrast, *Nr1d2* represses *Ppargc1a* when energy demand decreases [78]. *Fads1* is regulated similarly to *Ppargc1a* by circadian genes, specifically for fatty acid desaturation. *Adcy1* and *Adcy8* regulate cAMP levels during the active phase, which promotes lipolysis and fatty acid oxidation. Finally, *Ncoa2* activates genes that modulate the Clock-Bmal1 complex, including the periodic genes (*Per1*, *Per2* and *Per3*), cryptochrome genes (*Cry1* and *Cry2*), *Dbp* gene, Rev-erb genes (*Nr1d1* and *Nr1d2*), ROR genes (*Rora*, *Rorb* and *Rorg*) and metabolic genes (*Ppara*, *Nampt* and *Gys2*).

### 4.1. New pathways related to metabolism

The Boolean network suggests additional gene interactions, such as the pathway *Tef* --> *Fbxw11* --> *Dbp* --> *Pgr* --> *Ppargc1a*, which is not previously reported in the literature. This pathway implies that *Ppargc1a* is influenced not only by circadian genes, but also by genes like *Tef*, *Fbxw11* and *Pgr*. *Tef*, which regulates transcription via the TATA-box element, may modulate *Fbxw11*, a gene responsible for targeting proteins for degradation [79]. This interaction could influence *Dbp*, potentially modulating its expression. Studies suggest that *Dbp* could affect *Pgr* expression, acting as a signal transducer [80]. Moreover, *Pgr* is susceptible to daily regulation, particularly through *Dbp*, and is involved in interventions like hormonal therapies [81] and physiological regulation [82]. Finally, *Pgr* could modulate *Ppargc1a* expression, as similar hormonal effects, such as insulin or *Hif1a* in hypoxia, have been documented [83,84].

Another potential undocumented pathway is *Ppargc1a* --> *Ep300* --> *Ncoa2*. While the direct influence of *Ppargc1a* on *Ep300* has not been previously reported, the relationship from *Ep300* to *Ppargc1a* is well-documented, where *Ep300* acts as a co-activator, particularly in obesity and thermogenesis [83]. Notably, studies have shown that *Ppargc1a* can function as a co-activator [85], suggesting that, in this context, *Ppargc1a* may promote *Ep300* activity. Regarding *Ep300*’s activation of *Ncoa2*, this is supported by *Ep300*’s histone acetyltransferase (HAT) activity, which modifies chromatin structure, making it more accessible and facilitating the transcription of target genes through nuclear receptors. This mechanism is particularly relevant for the p160 co-activator family, to which *Ncoa2* belongs [86].

### 4.2. Obesity effects

Our Boolean model also revealed that obesity leads to down-regulation of key circadian genes, *Bmal1* and *Clock*, which may impact several metabolic processes, including glucose homeostasis and insulin response [87,88]. Interestingly, *Bmal1* influences *Fads1*, which in turn affects *Clock*, and together, *Bmal1* and *Clock* regulate lipid metabolism through E-transcriptional activation [87]. This suggests bidirectional interactions between lipid metabolism and circadian rhythms under normal conditions. In obesity, the downregulation of both *Bmal1* and *Fads1* leads to impaired coordination between *Bmal1* and *Clock*, further compromising lipid metabolism regulation [89]. Additionally, this disruption may worsen over time, as evidenced by recent findings linking circadian dysfunction in omental fat with obesity [90] and the presence of single nucleotide polymorphisms in the *Clock* gene has been associated with morbid obesity [91]. This circular pathway appears to be previously undocumented.

### 4.3. Limitations of this study

The main limitation of this study is the use of the Boolean model, which simplifies by reducing it to two states, failing to capture the full complexity of gene interactions. As previously mentioned, this approach offers a tractable framework for modeling gene-level interactions, its binary “on/off” cannot capture the graded or temporal nuances of gene regulation. Despite this limitation, the model used provides valuable insights that could be expanded upon with more detailed models, such as probabilistic or differential-equation models.

Our analysis was also constrained by the limited gene set – primarily lipid metabolism- derived from a single cortical transcriptome dataset. This small size and cortex-only focus may limit the generalizability of our findings. Cross-validating our results using larger, independent obesity-related datasets (e.g., hypothalamus, liver, adipose tissue etc) will expand the network to include additional genes impacted by these analyses.

Finally, the GENIE3 algorithm infers directed edges from expression data alone and may miss well-documented interactions (e.g., the Clock-Bmal1 feedback loop) when statistical support is weak. Future studies should integrate curated interaction databases and literature-derived edges to reinforce and refine network topology.

## 5. Conclusions

Our study on circadian rhythms and obesity has revealed significant interactions, suggesting that disruptions in these rhythms worsen metabolic dysfunctions. This finding highlights the importance of maintaining regular circadian rhythms to mitigate the health risks associated with obesity.

The analysis of DEGs linked to circadian rhythms provided valuable insight into the functional mechanism underlying obesity. Enrichment analysis identified key DEGs that help explain how obesity alters circadian functions, contributing to conditions like metabolic syndrome and its comorbidities. Our Boolean model effectively captured this complex interaction; however, given the complexity of these interactions, further studies are needed to better understand the causal relationships between circadian rhythms disturbances and obesity.

## Supporting information

S1 Fig Preparation of the transcriptomes: (A) normalization and (B) representation based on the mean-standard deviation model. (DOCX)

S2 Fig Iterations of the genes Dbp, Fbxw11, Nrg1 and Pgr to produce the two states of attractor 40. (DOCX)
