# Supplementary material for: Exploring the interplay between circadian rhythms and obesity: A Boolean network approach to understanding metabolic dysregulation
Source: PLoS One. 2025 Sep 9;20(9):e0331218. doi: 10.1371/journal.pone.0331218 (PMC12419585; doi:10.1371/journal.pone.0331218)
Supplement: S1 Fig — (DOCX) [file pone.0331218.s001.docx]

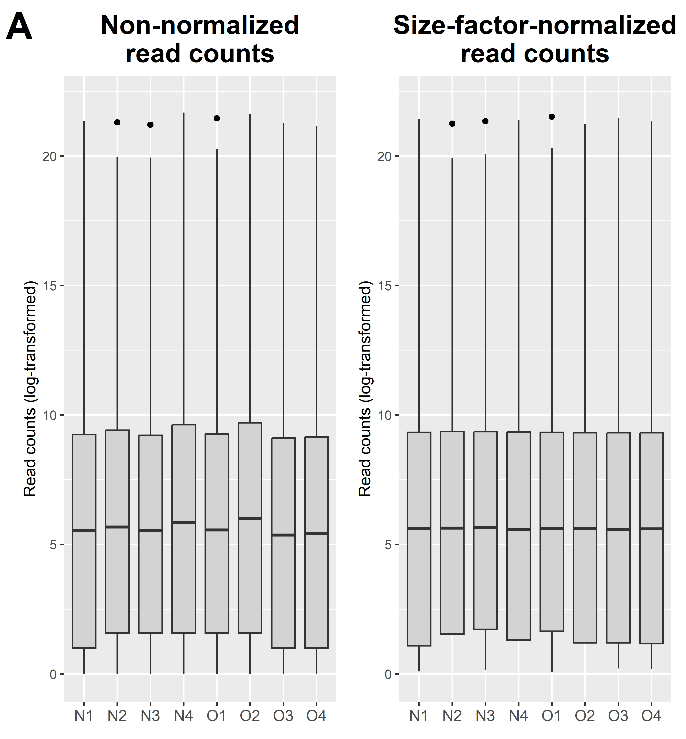

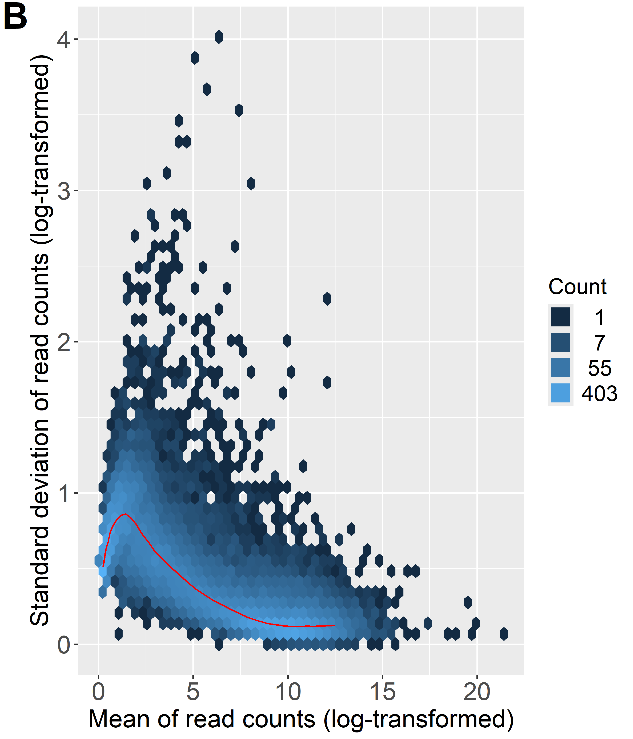


Figure S1: Preparation of the transcriptomes: (A) normalization and (B) representation based on the mean-standard deviation model.
