# Supplementary figures and images for: Exploring the interplay between circadian rhythms and obesity: A Boolean network approach to understanding metabolic dysregulation
Source: PLoS One. 2025 Sep 9;20(9):e0331218. doi: 10.1371/journal.pone.0331218 (PMC12419585; doi:10.1371/journal.pone.0331218)

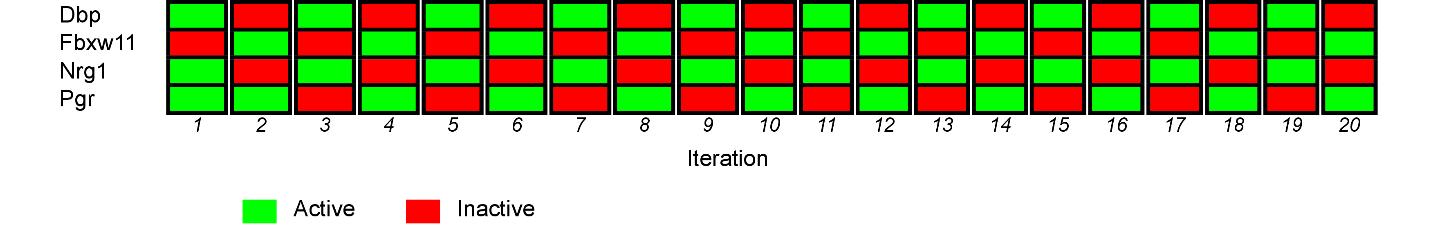


Figure S2: Iterations of the genes *Dbp*, *Fbxw11*, *Nrg1* and *Pgr* to produce the two states of attractor 40.

Supplement: S2 Fig — (DOCX) [file pone.0331218.s002.docx]
